# Collections from the Unpublished Medical Writings of the Late Dr. Parry. Vol I

**Published:** 1826-01

**Authors:** 


*Collections from the unpublished Medical Writings of the late* CALEB HILLIER PARRY, M.D. F.R.S. &c. Vol. I. pp. 590, Royal 8vo. Underwoods, June, 1825.

"Even in our ashes live their wonted fires!"

THERE is a melancholy pleasure in perusing the posthumous works of the "illustrious dead." The fate of Dr. Parry's writings is like—and unlike that of the Sybilline leaves. They were appreciated at first—and they will be ardently sought after at last. We talk of a man's *immortality*, without reference to a future existence—for that is not likely to be in this planet. The *immortality* of heroes, poets, philosophers, legislators, and physicians, consists in the *memory* of their actions and their writings. Medicine has some reason to be proud of the early immortality conferred on some of its professors. Without going into the regions of fable or poetry, the works of Hippocrates, Celsus, Galen, and others, afford prospects of the immortality in question, to those who distinguish themselves in the healing art. But although the field of our ignorance—that is, of what remains to be ascertained, progressively expands in proportion as we advance, yet it must be confessed that the limits of our powers appear narrower and narrower as we exert those powers—thus curtailing the arena of the medical philosopher's ambition within a very small span indeed! In anatomy, the basis of our knowledge, there is but little more to discover. In physiology there has been very little ascertained, except the fallacy of what was once supposed to be truth. What do we know of digestion, secretion, assimilation, sanguification, or any of the processes hourly going forward within this living machine?—Nothing, but that they exist! In pathology and therapeutics we have advanced many steps, and may yet advance many more. We may yet acquire much more knowledge than we now possess of the action of morbific and medicinal agents on the human frame. This knowledge is at once the most curious, and the most useful of any to which the mind of the medical practitioner can be directed. It is evidently on this department of medical science that the great mind of the elder Parry was principally employed. Alas! that he should have been cut off before he completed his task! His "Elements of Pathology and Therapeutics" appeared like the poitico of a magnificent temple, exciting sanguine hopes and ardent curiosity. His posthumous works disclose an immense space, covered with materials for the vast design, in a state of greater or less forwardness, and too plainly indicating that the giant arm of the architect was paralysed at the moment he thought himself prepared for the erection of the edifice. Here, then, is an ample amphitheatre of recent, or we might say, antidated ruins, where other architects may find abundance of valuable materials for other —but humbler constructions! How truly prophetic was the following sentence, written by Dr. Parry himself. "I perceived that the time which it would occupy was inconsistent with the incessant pursuits of a toilsome profession, and that, so far from my having any reasonable chance of reaching the end of my proposed journey, my sun would probably set, while I was yet far distant on the road." 6. That sun is set —never to rise again! But the rays of light which he emitted, while above our horizon, will illuminate the dark and dreary path of many a traveller on the same road.

We are far from being blind idolaters of this sun of medical science, or considering him as without a macula. Like all human beings, he was prone to error. Like every genius, he had a dash of enthusiasm in his composition, which, while it tends to brighten some of the mental faculties, has unquestionably the effect of adumbrating others. In our review of his former work, we pointed out, what we conceived to be, some most important and fundamental errors, especially as related to the nervous system; and we shall have occasion, in the analysis of his posthumous productions, to differ from him on several occasions. This we shall do, not with the view of detracting from the merits of the illustrious dead, but with the hope of rendering his labours more useful to his successors, and to humanity in general.

We think Dr. Parry has committed an error in the very first page of his preface, where he attributes the retardation of medical science rather to the perverseness of the human mind than to the difficulty of the subject. We admit that the *former* has considerable influence, but nothing when compared with the *latter*.

"The knowledge of mechanics is already so extended as to afford us many fixed and invariable laws; and chemical facts are daily presenting themselves to us in numbers so great, as soon necessarily to produce some determinate principles. It must, therefore, at first sight, afford cause for astonishment and regret that Medicine, the most important branch of natural knowledge, which has for its object the prolongation of animal life, and the preservation of the bodily and mental powers, should have been left in the race so far behind its sister sciences. A little observation will, however, point out the great cause of this deficiency. The laws which govern the human frame might doubtless be experimentally ascertained with nearly as much precision as those of mechanics and chemistry, were it not for the intervention of another part of the compound which we would scrutinize. This is Mind, nurtured in prejudice and error, and therefore selfish; incommunicative and inaccurate, yet proudly expectant; at once capricious, irresolute, obstinate, and vindictive; suspicious; offended by reasoning, and averse to truth, yet credulous, and servilely grateful for being duped; equally intolerant of present inconvenience, and prone to present gratification. Examples of the difficulties arising from this source, which I have experienced during a professional practice of nearly forty years, crowd so forcibly and in such numbers on my memory, that to enumerate only a small part of them, would be to write the severest satire on the follies and vices of mankind." 2.

Our author admits the difficulty and the extent of medical science, which, taken in all its relations, comprehends a knowledge of the general laws of nature, as well as the particular laws of the animal economy. Thus the accomplished physician ought to be acquainted with the properties of number and figure —the laws of mechanics and hydraulics—the general principles of botany and chemistry—the anatomy of man and of other animals—and metaphysics. When he has acquired all these he is only at the threshold of his profession. He must study the almost endless structures and functions of the human machine—their various ties and nice dependencies—their movements and affections in a state of health—and the signs and consequences of their deviation from that state. Lastly, he must inform himself of the causes which disturb, and the remedies which restore the healthy functions, whether of mind or body.

"If the science of medicine be thus important and difficult, one might reasonably expect that it would, on its own account, be honoured among mankind, and its interests assiduously promoted. Above all, one would presume, that those who worthily profess it, would hold, among the orders of society, a rank precisely proportioned to the civilization of the country in which they lived.

"What, then, will posterity conclude of the barbarism of a country, which, at the beginning of the 19th century, places medical graduates in the very lowest rank of privileged society? *What will they think of men, chosen from an important branch of the legislative body, who could openly assert the inexpediency of employing physicians to superintend the health of the brave defenders of their country?* It requires no strong powers of observation to see the tendency of such principles to discourage the scientific pursuit of medicine, and to debase it to the level of a mere mechanical art, capable of being practised by the meanest and most sordid of mankind." 5.

We hardly know what is alluded to in the passage which we have printed in Italics. Certain it is that no imputation of the kind attaches to the times we now live in. If Dr. Parry alludes to the revolution which many years ago placed the care of military hospitals in the hands of military physicians and surgeons, he here presents a melancholy instance of that "prejudice and error" which he so much deplores. The civilian physician would now be ashamed to throw out the slightest hint respecting the injustice of the decree which rescued our meritorious medical officers of the King's service from the degrading yoke under which they were placed by a "proudly expectant, capricious, irresolute, obstinate, vindictive, suspicious, and servilely grateful" policy. But those days are over. The medical officers of our fleets and armies fear no comparison with their brethren in civil life—and, therefore, need no vindication of their professional character in this place. As to the degradation of medical rank generally, we see nothing of it but what arises from the self-degradation of individuals. Medical men, who conduct themselves properly, and practise their profession with honour, zeal, and liberality, will always obtain their due level in the estimation of society, and receive a full share of that respect which is paid to integrity and merit—and which *ought not* to be paid to any thing else.

But when we see the mean and base arts which are daily practised by some of even the higher orders of medical men, to gain popularity and fill their pockets, we cannot wonder that the more intelligent and discerning portion of the community should occasionally detect such manœuvres, and thence form an unfavourable opinion of an otherwise learned and liberal profession. This is the great source of mischief—and we fear it will long continue so!

In a preface of 55 pages, Dr. Parry descants on education, perception, reasoning, cause and effect, succession of phenomena, various sources of error in our observations, predisposition to disease, and numerous other topics, which it would be impossible, and perhaps unprofitable, to notice in this place. His prefatory observations, however, will be read both with interest and advantage by all who take up the book—and we hope all will take it up. From this portion we are tempted to make one extract.

"It cannot, however, be denied that, in this respect, the profession of medicine labours under peculiar disadvantages. The very multiplication of the opportunities of knowledge so harrasses and fatigues by the incessant practice of the art, as often to afford little leisure or inclination to cultivate and extend the science. If to this rule there occur some few exceptions, they depend not on any superiority of original talents, but on early habits of mental application, on the force of motives, on the felicity of local situation, and on the capacity of the body to endure privation and labour without suffering that languor, which would impair the energy of the mind.

"The business of Man is not merely to eat, to drink, to sleep, to enjoy sensual pleasures, and then to lay himself down and die. Exclusively of eternal concerns, every human being should have some one great and laudable end in life, which should constitute his chief motive to action, and to which, therefore, all his other occupations should be subservient. Habits of this kind having been long formed, whatever may be the nature of the object in view, or however difficult its attainment, the pursuit is no longer painful. On the contrary, the mind associates it with all other trains of thought, reluctantly wanders from it, and returns to it with delight as to its native home.

"Feelings like these, which have long made my professional pursuits my greatest pleasure, aided by the wish of emulating some great professional names, and by a strong desire that the world may be the better for me after I shall have left it for ever, have supported me under the privation of domestic and social gratifications, and under exertions incessantly pursued through sickness, sorrow, and pain." 47.

"The great Book of Nature, which is alike open to all, and is 'incapable of deceiving,' I have hourly read, and I trust not wholly in vain. During the first twelve or fourteen years of my professional life,

I recorded almost every case which occurred to me either in private practice, or in the chief conduct of an extensive Charity. When afterwards the multiplication of common examples seemed to me an unnecessary waste of inestimable time, which might be much more profitably employed, I contented myself with the more useful task of recording chiefly such cases, or, on a few occasions, such particular circumstances only of cases, as led to the establishment of principles. This I have generally done on the spot, or rarely deferred beyond the day of observation, always rejecting what, on repeated and varied inquiry, I have not been able fully to verify.

"Whatever inferences from phenomena have suggested themselves to me, I have immediately noted down, and afterwards carefully examined on all sides and in every light. By this method, which I strenuously recommended to all persons engaged in scientific pursuits, whether physical or moral, I have often been able to ascertain the order of phenomena, and to catch new links, which have gone some way towards completing the whole chain of causes and effects." 48.

Who can peruse the above passages without heaving the sigh of regret, that such a man should not have been spared to arrange and finish a work constructed of such inestimable materials!

Dr. Parry ends his preface with a severe Philippic against Brown and his doctrines, than which last, he thinks, no set of medical rules "has been more destructive of human health and morals, and, therefore, of human happiness, from the days of Hippocrates to the present time." We believe that, for a few years in this country, and for many years on the Continent, the Brunonian doctrines destroyed a considerable number of sick people annually. But we question whether the short reign of his theories produced any demoralising effect on society at large, in the way of intemperance, (the only way in which they could act) since it is universally allowed that, for the last twenty years, the upper classes have become so progressively weaned from the immoderate use of fermented liquors, that it is now a phenomenon to see a gentleman drunk.

We have compared the matter of the present volume to a mass of materials accumulated and prepared for the erection of a vast building—or to a heap of recent ruins strewed in disorder on the ground. The comparison is literally just. There seems to be no dependence nor connexion between the parts. They are all insulated notes, memoranda, or reflexions, without any apparent attempt to arrange them into shape or order. This circumstance renders it exceedingly difficult—nay, impossible, to exhibit any view of the work which may not appear, like the original, an unconnected succession of rude, though valuable materials. We shall pass entirely over the anatomical and physiological portions of the volume, occupying 140 pages, commencing our analysis and extracts with a section headed "IRREGULAR DETERMINATION."

I. This is a subject much talked of but little understood at present. We see these irregular determinations, particularly in weak and irritable habits; but whether they are the primary, or only the intermediate causes of disease, may admit of doubt.

"The causes," says Dr. Parry, "of irregular determination are of two kinds; *First*, those which immediately destroy or change the balance of circulation, 1st, by diminishing the flow of blood to one part and so increasing it to another, as throwing more blood to the head by cold applied to the feet; 2dly, by weakening the propelling power of the vessels in a part, and so producing accumulations in it. And, *secondly*, those which excite increased action of the heart, and momentum of the blood, which then acts more especially on parts predisposed, producing in the brain the usual effects of increased determination, in other parts inflammation, increased discharges of blood or serum, as hæmorrhages and dropsies, sweating, &c.

"*Order of Facts in Irregular Determination.*—Whatever may be the reason, whether it depend on any thing in the state of the blood itself, or of the vessels, or whatever law regulates the circulation, it seems certain that gout, epilepsy, and various other diseases of irregular circulation, arise not immediately from increased determination, but after that increase has subsisted some time, or, according to the Brunonian language, from indirect debility." 143.

Thus, when the action of the heart is increased in certain constitutions from any cause, as flatus in the stomach, mental agitation, &c. a rush of blood may take place, beyond the due balance, to the head, lungs, or some other part, and hysteria, mental alienation, epilepsy, asthma, &c. may follow.

II. *Inflammation*. It is difficult to ascertain the proximate cause of this affection. The first phenomena, Dr. P. avers to be—increased momentum, (that is, increased velocity with the same quantity)—equal velocity with increased quantity—or, diminished velocity with increased quantity.

"From all which has been said, it is highly probable, first, that in a state of local inflammation there is a morbid determination of blood to the capillary vessels of the part affected, which consists in an increased momentum in those vessels which carried blood in the healthy state, or in the intromission of blood where they were previously void of it; but, secondly, that in a more advanced stage of the disease, a slower circulation and a diminished impetus, or even a total stagnation, of that fluid may ensue; and, thirdly, that then will follow the various effects already specified, chiefly intended either for the cure, or renovation of the affected part.

"What it is that especially determines either of these several effects of inflammation has not hitherto been discovered." 147.

III. Our author criticises, though in a very unconnected manner, certain opinions of Bichat, and then abruptly starts to the subject of fever. He has not been able to throw much light on the theory of this important class of diseases. He appears to think, and not without reason, that the paroxysm of fever, say an ague, is a salutary effort of the constitution "to restore circulation, or to get rid of superfluous blood in a part."

The following case is curious.

"Dr. C. aged upwards of 60, contracted in Lincolnshire an ague of the pure Tertian type, for which he tried, in vain, all the remedies that could be suggested by his medical brethren in the metropolis and elsewhere. Among other medicines, he had taken arsenic so as to produce paralysis of the four extremities. At length he came under my care at Bath.

"I also employed every method which I could devise, or learn from others, but altogether without success. As long as the best bark remained on his stomach in substance, the disorder ceased; but at the end of three or four days, he always vomited up, not only the bark itself, but all the food of every kind which he took during its use. He was therefore obliged to discontinue it; and in a few hours the paroxysm returned as before. Thus, at the end of two years, he died.

"On examination of his body, the integuments of the thorax and abdomen had on them at least three-quarters of an inch of fat.

"The mesentery was very much thickened, and the omentum contracted, moderately fat, and adhering to various parts. The mesentery and peritonæal coat of the small intestines and of the parietes of the abdomen, were thickly studded with small scirrhous glands. The liver was sound, but strongly adhering on its anterior and superior part to the peritonæum. That portion of the peritonæum which covered the colon was free from disease. The spleen was in a natural state, as were the intestines and stomach. The latter contained in it about a pint of brown glairy fluid, similar to what was usually rejected by vomiting. In the abdomen, there was nearly a quart of serous fluid.

"In the lungs, there were a few tubercles, not in a state of inflammation. In the thorax, a pint of fluid. The heart was very fat; and all its valves, together with the coronaries, and adjacent large vessels, free from ossification and all other disease. [Jan. 17, 1802." 157.

In the same page Dr. Parry hazards the following conjecture. Speaking of typhus and scarlatina, he observes :—

"A very large proportion, I think I may say full four-fifths, of the cases of Typhus which I have visited among the better classes of people, have been either in lone houses in elevated situations, or, if in towns, in the most airy outskirts. The latter also has been in a still larger proportion true with regard to Scarlatina. I cannot help therefore thinking that some state of the air, as its weight or temperature, must have a considerable share in producing these diseases." 157.

Now we really know not what grounds Dr. Parry can have for supposing that typhus and scarlatina happening in the "airy outskirts" of a town should be owing to atmospheric influence rather than to the specific poison which is known to produce them, (at least one of them, scarlatina) in the way of contagion.

Does not the poison of variola become diffused in the atmosphere and, under certain conditions of that medium, give rise to epidemic small-pox ? Why should not the poison of typhus and scarlatina be liable to the same laws ?

IV. Speaking of the connexion of fever with local disease, our author remarks as follows: "When fevers come on, *accompanying local affections*, it is not easy to decide whether they are symptoms or causes. They may be efforts to relieve the apparent local disease, or the cause of it—or they may be to relieve some non-apparent disorder, and produce local diseases in other parts." The meaning of this passage is not very clear ; but it is evident that our able author is not an advocate for fever being the result of local affection.

"*Beneficial Effects of late Bleeding in Fever.*—In Master C. aged nine, on the sixteenth day of a continued fever, with little preternatural heat of skin, a pulse between 120 and 136, and constant pain on the left side of the head, followed by delirium, subsultus tendinum, with speechlessness and almost constant stupor, the stools procured by calomel and salts were like colourless jelly much diluted, and the pupils contracted irregularly and unequally on the admission of light. Under these deplorable circumstances, which existed in spite of very active previous measures of depletion, eight ounces of blood were taken in the evening from the left temporal artery ; and as after this operation the child was evidently less delirious, and the pupils contracted equally and readily, the pulse remaining sufficiently full, the same quantity of blood was taken from the same artery the next morning. At the same time twelve grains of rhubarb were given.

"And now two phenomena occurred, from which I entertained considerable hopes of a favourable event. The serum of the blood last taken had somewhat of that milky appearance which is observed to take place in that of blood drawn during tolerable health soon after a meal: and as this appearance is generally supposed to arise from an admixture of newly formed chyle, its existence in the present instance was an indication of healthy absorption, and consequently of an interruption of the destructive processes of fever. In the afternoon there was also, for the first time for many days, a fæculent and consistent stool, of a dark brown colour, which was a further evidence of a proper action of the alimentary canal, and of a healthy performance of the hepatic function." 159.

No wine or other cordial was given through the whole course of this disease.

V. Dr. Parry next relates some particulars of a case of hectic relieved by hydropic effusions. The patient was a lady aged 30, who, after long and violent pain above the pubes, without swelling, began to discharge purulent matter with her stools. This state continued for many weeks, with constant hectic fever, night sweats, and emaciation, her pulse ranging from 120 to 136. In the course of the complaint, the subcutaneous glands swelled and inflamed, in various parts of the body, but gradually subsided. There was vertigo, with occasional delirium. By degrees a dropsical swelling took place in the abdomen, and as this increased, the pulse fell, till at length it came down to 96, with a proportionate diminution of the heat and nightsweats. After some time, the hydropic swelling extended itself to the lower, and even the upper extremities. There was reason, from the shortness of breath, to suppose also that there was effusion in the chest. The purulent discharge from the bowels, to the amount of half an ounce or an ounce, continued every three or four days. The pulse fell even below the natural range, and the night-sweats, vertigo, and delirium, entirely disappeared.

" Now, however, another conspicuous and notable change of circumstances occurred. The œdematous swelling of the hands began to abate; and on the 24th, in the morning, the pulse was 72, and irregular, and the respiration 36. On the 25th, the œdema still decreasing, and the breath being somewhat better, with considerable dry cough, the night having been restless from pains in various parts, and the urine small in quantity, high-coloured, and with a copious farinaceous sediment, the pulse rose to 104. On the 26th, it was in the same state. The swellings still continued decreasing, and with them the powers of life till the 4th of March; when, the former being nearly gone, the pulse 90, and the respiration 52, she soon afterwards expired." 161.

VI. *Erysipelas.* Our author appears fond of the application of cold washes externally to the skin of the face and head, in cases of acute erysipelas, where there is great determination to the brain, quick incoherent talking, violent agitation, &c. &c. "The application which I employ, with immediate relief from the most violent burning pain, is one part of common spirit of hartshorn to three of cold water, on rags several times doubled and constantly renewed on the part."

VII. *Affections of the Skin.* We believe there is much truth in the following observations of our intelligent author.

"I think it also probable, that the skin is the seat of disorder in various complaints that are usually called muscular. In nervous women we often observe obstinate and troublesome pains about the sides under the breasts, accompanied with more or less of tenderness on pressure, and indisposing them to lie on the affected side, though without marks of fever or inflammation. Often in coughs, the sides and belly are very sore; so are all parts of the body after uncommon exertions of the muscles; the calves of the legs and other parts in cramps. In the case of nervous women before mentioned, the pain seems to be accompanied with even cramp or contraction of the muscles. Now it appears to me that these are chiefly affections of the skin, which we know is a very tender part, while the muscles are much less sensible, and which is so connected with the muscles by cellular membranes and blood-vessels, that it is easily affected by all preternatural motions of the muscles themselves." 167.

VIII. *Scarlatina.* Nothing, Dr. Parry thinks, can afford stronger evidence that dropsy is owing to an increased momentum, that what often, nay usually, occurs in scarlatina. We confess that the dropsical effusion, succeeding scarlatina, is to us no proof that the cause of dropsy in general is of an inflammatory nature. We are well aware that inflammation of the serous membranes very frequently gives rise to hydropic effusions in the cavities; but we are equally convinced that ascites and anasarca often take place where there is any thing but an inflammatory diathesis in the system.

"When dropsy is associated with large artificial hæmorrhages, I observe that, as in the case of hæmorrhages which are spontaneous, it does not usually accompany them, but comes on after they have ceased; and I have concluded that it is the effect of nutriment absorbed too suddenly for the relative state of the vessels, which therefore strive, if I may be allowed the expression, to get rid of it by every possible outlet. This principle is well illustrated by the following case. Mr. H. at the middle period of life, of great and just professional reputation in this city, and about a year before confined for many months to his bed with acute general gout, was attended by Mr. G. Norman and myself, for a violent inflammation in one eye, accompanied with great pain and fever, and threatening the speedy loss of that organ. In the space of four days, twenty-five ounces of blood were taken from the temporal artery on the same side, and seventy-five ounces from the arm, many leeches were applied to the temple, and the vessels of the conjunctiva were frequently scarified. From these powerful means, aided by purging, nauseating doses of ipecacuanha, and iced water, almost constantly applied to the forehead and crown of the head, the disease in a few days disappeared; but suffered a relapse, in consequence of his leaving his bedroom too suddenly, and taking a meal of animal food. It was, however, once more removed by another bloodletting to the amount of twenty-four ounces. Shortly afterwards he returned to his professional pursuits, and for three days dined only on chicken. His legs now began to be affected with œdematous swelling; and as it was considered that this affection was owing to too sudden a resumption of full diet, he left off animal food, and purged himself; in consequence of which, at the end of three days more, the œdema vanished, and he now, after an interval of many months, continues to enjoy a degree of health and strength, to which for several years before he had been a stranger." 186.

IX. *Dropsy a Curative Process.* In illustration of this subject, Dr. Parry relates the case of Lord G———, (we presume Lord Gardiner) who came to Bath in 1808, being then in the 67th year of his age, having been appointed to the command of the Channel fleet the year before, in which situation he was in a constant state of anxiety respecting the faithful discharge of his duty.

" On his arrival at Bath, Sept. 25, he was labouring under difficult and hurried respiration, so that not only walking, but even speaking was uneasy to him. He was obliged to lie with his head and shoulders raised, and could not sleep on either side, but had no cough. His urine did not amount to three-quarters of a pint in 24 hours, was of a darker colour than porter, and had a copious sediment. His ankles were not swelled; he had no pain or fulness in the region of the liver, or any other part of the abdomen, and no yellowness or discolouration of the skin. His pulse was 84 in a minute, and his respiration 32. His tongue was rather dry, but not furred. He was much emaciated, and had an almost total loss of appetite. He had been treated with purgatives and chalybeates.

" The remedies now ordered were a grain of quicksilver rubbed down with manna, and the same quantity of dried squill, twice a day, and twice or thrice a day an effervescing draught of citrate of potash, with fifteen drops of Mr. Tickell's preparation of ether. In three or four days the urine increased to three pints in the twenty-four hours, and Lord G's health was greatly mended.

" The pulse being now of the natural standard, and the appetite continuing bad, Lord G. was desirous of drinking the Bath water, from which, on a former occasion, he had derived considerable benefit. The Cross Bath water was given at first once, and then twice a day, in the quantity of only four ounces at each dose, and the other remedies were to a certain extent continued. In a very few days the pulse began to quicken, the urine to decrease, and the breathing to become more laborious, and that without any improvement of the appetite.

"The Bath water was now omitted, and the medicines were given in the full dose as before. The urine was again soon restored, the breath amended, and the pulse reduced nearly to its natural state." 189.

Again Lord G's desire for drinking the Bath waters returned, and again it was indulged; but with still worse effects than before. Fever, paucity of urine, dyspnœa, were again, in a few days excited, and cough was superadded, accompanied by spitting of blood, threatening instant suffocation. Squills and effervescing draughts were substituted for the Bath waters, with the effect of once more removing the fever and pulmonary affections, and of restoring the urinary secretion. After some weeks passed in an improving condition, the stools were observed to become pale, and the feet somewhat œdematous in the evening, the œdema gradually extending up to the scrotum. The mercurial course was again commenced, but without any other effect than that of keeping the bowels open. At this time, although Lord G. was taking six grains of squill daily, he began to relish his food better than at any former period; soon after which his belly began to swell, and in about a fortnight, fluctuation was evident. As the abdominal swelling increased, that of the legs somewhat diminished, and the appetite mended. The sleep and facility of breathing also improved. In this state Lord G. continued several weeks, with little increase of emaciation or weakness. Mercurial ointment was daily rubbed on the abdomen for a month, at length occasioning some soreness of the mouth, when it was discontinued. Various remedies were tried till the middle of December, when, after some days' absence, Dr. Parry was surprized one morning to find the abdominal fluctuation absent, although there had been no particular evacuation by any of the ostensible outlets. But now he was observed to be labouring under fever, with restless nights. He had been taking a combination of quicksilver with squill and æther, which was now continued, with the addition of laudanum. By the 25th December his urine was increased to nearly a gallon daily, which afterwards varied from one to two quarts *per diem*. On the 31st December his pulse was 80, regular, and soft—his respiration 32, and he seemed to sleep quietly. He died, however, in the evening, without a struggle. No dissection is given. But there is little doubt that he died of hydrothorax.

*Cure of Dropsy*. Dr. Parry has long considered the operation of remedies in curing dropsies, as influencing and removing their cause, whatever that may be—more especially disordered action of the heart or arterial system. Of this state, deficiency of urine, is a usual, but not a constant effect.

"For I have seen at least ten cases, at various ages, from fifteen to sixty, of dropsy, chiefly anasarca ending in hydrothorax, in which the quantity of urine has spontaneously been from the beginning, till two or three days before death, fully equal to that which was natural, as for example, from two to four pints daily, clear, and even sometimes pale, and yet the patients have all died. Nay, I have never seen a patient recover under these circumstances of a natural state of urine.

"On the other hand, I have known many examples of patients labouring under dropsy of different kinds, with defective and high-coloured urine, who have perfectly recovered, all the effused water having been absorbed; and yet the remedies producing the cure did no more than just restore the urine to its healthy quantity and colour.

"The connexion of deficient urine is, therefore, only an effect, and by no means a cause." 192.

*Bleeding in Dropsy.* About the year 1788, Dr. Parry was called to a celebrated horse-dealer, between 50 and 60 years of age, who had been a great spirit-drinker, and laboured under diseased liver, with ascites, anasarca, a quick pulse, extreme paucity of urine, which was high-coloured and sedimentous. A great variety of remedies having been tried in vain, Dr. P. desired venesection to eight or ten ounces to be performed, which exhibited the inflammatory crust. Without the aid of any other remedy, the pulse was reduced in frequency, and the urine during the next 24 hours was quadrupled in quantity, becoming clear and pale. A Brunonian physician being now joined in attendance, the depletory system was not followed up.

The following case, we think, is worthy of record, as shewing how long ago Dr. Parry anticipated doctrines which are considered of much more recent date.

"Col. G. O. a short, rather corpulent man, between fifty and sixty years of age, who had been a free liver, and subject to frequent fits of the gout, became my patient in consequence of great œdematous swellings in his legs, paucity of urine, and total loss of appetite, for which, as they were supposed to be connected with a gouty diathesis, he was sent to Bath for the use of the waters. At my first visit, I found him labouring under these symptoms, to which were added a sense of great heaviness in his head, a flushing of his face, disturbed sleep, an occasional stertorous inspiration while he was awake, depressed spirits, a very dry tongue, and full, strong, and a rather quick pulse.

"At this time, Aug. 1790, I was young in my profession, but not altogether unobservant of the facts which presented themselves to me, and as it had happened to me to see more than one case extremely similar to that which I have described, in which the patient either had afterwards epilepsy, or was relieved by copious hæmorrhage from the nose, I ventured to predict that one or other of these events would occur to Col. O., unless they were prevented by a timely artificial evacuation of blood.

" It will readily be conceived what impression this proposition, at that period of medical knowledge, made on the minds of the patient and his friends. What! bleed a man at an advanced period of life, of a gouty habit, in such a state of debility, actually labouring under dropsy, and who clearly wanted blood, rather than suffered from a superfluity of it! Never surely was there so wild and extravagant a proposition!

" Notwithstanding these objections, I persevered, and was fortunate enough to gain my point. Ten ounces of blood were taken away by cupping. The patient felt immediate relief of all the more important symptoms. The weight in his head and stertorous inspiration were removed, the tongue became less dry, the pulse softer and slower, he had that night many hours of comfortable and refreshing sleep, and waked with some appetite.

" But though the remedy was considerably beneficial, it was insufficient for the purposes of the constitution, for on the following day he was seized with a spontaneous bleeding at the nose, which was very copious, and in a few days no symptom of indisposition remained." 195.

Several other cases, of a similar kind, are related, and also instances of relief from anasarca, in consequence of spontaneous hæmorrhage.

X. Under the head of "*tendency of stimulants to produce effusions*, especially when given during convalescence," Dr. Parry gives a case in illustration, the outline of which may not be without some use to young practitioners.

A gentleman, ætat. 19, much addicted to field-sports, to wine, and to women—whose father died of hepatic abscess—became affected with syphilis in the autumn, for which he underwent a mercurial course. Having exposed himself to cold, while under the influence of mercury, he became affected with cough, accompanied by fever, but did not refrain from his usual indulgences, till a decided pleuritic inflammation became developed. No active measures were at first employed, but afterwards copious depletion and other antiphlogistic methods were adopted. The symptoms of active inflammation had subsided, when Dr. P. first visited him. His pulse was still considerably above 100 in the evenings, but soft and weak—skin cool—tongue moist and clean. He could not take a deep inspiration easily. Walking produced breathlessness—"and when he lay down his respiration was considerably incommoded by the sensation of a heavy weight falling in his chest to that side on which he happened to lie." Yet he had no cough—countenance pale—urine high-coloured and defective—legs and feet œdematous. Some slight tenderness in the right hypochondrium—appetite moderate—stools pale. Conceiving that hydropic effusion had taken place in the chest, our author recommended a continuance of the antiphlogistic diet, together with squills, digitalis, and saline draughts, with occasional doses of calomel as a purgative. In a few days, the pulse came down to 72, even late in the evening—the urine became natural in colour and quantity—and the breathing improved. He thus went on improving, till some over-anxious friends, "fearful of debility and other visionary evils," persuaded him to take animal food and wine. In a few days after this change of regimen, his pulse again quickened—his belly began to swell—and fluctuation soon became evident!—Rigid abstinence was again enjoined, and squills and calomel prescribed. He once more mended, and eventually was restored to perfect health.

XI. *Inflammatory Petechiæ.* It is well known that Dr. Parry always considered the petechiæ hæmorrhagicæ as inflammatory. In this portion of the volume he relates several cases illustrative of his doctrines, of which we shall only select one, as a specimen.

" *Inflammatory Petechiæ.*—J. B. aged twenty-four, had for three or four years been subject to pain of the lower extremities, and chiefly of the ankles, which was occasionally relieved by topical œdematous swellings. About the beginning of March, 1814, he was seized with a great increase of the pain, which affected the left foot across the instep, accompanied with swelling and redness, great aggravation on moving the part, but little soreness to the touch. Neither the pain nor swelling affected any other part. The same night there came out spots of a petechial kind, of a dark red and sometimes purple colour, roundish, and of different sizes. They first affected both thighs, then extended themselves to the legs and feet. The thighs and legs were not considerably sore, but were stiff, and as it were benumbed, when he attempted to walk. His appetite for animal food was diminished; his pulse was somewhat quickened, he was thirsty, and his rest was disturbed. His urine was also high-coloured, with a deal of sediment.

" In this state he obtained some temporary benefit from purging. But the complaint returning with increased violence, he was blooded on the 25th of March, to the amount of ten or twelve ounces. The blood had all the appearances which occur in that of pleuritic patients; and at the end of three days the chief part of the disorder was removed.

" About this time he became very hoarse, with a considerable cough and soreness of the throat. On the 5th of April his pulse was natural, his skin cool, his tongue clean, his appetite good. He was, also, free from all pain and swelling of the foot and leg. He still, however, continued extremely hoarse, and a broad damask-coloured spot, of an irregular shape, occasionally appeared on the leg. I ordered another bleeding on the 7th, to the amount of twelve ounces. The blood was in precisely the same state as the former.

"On the 15th, no return of pain, swelling, or petechial spots had taken place. The hoarseness was almost gone; his pulse was slow and soft, and he was in other respects perfectly free from disease." 220.

XII. *Acute Rheumatism.* The following has been Dr. Parry's experience of the effects of bark in this disease. In one instance, a patient who had had several successive paroxysms of rheumatic inflammation lasting several days, and leaving a tolerably perfect intermission, the "bark did certainly appear to cure the patient, after the failure of sudorifics and other remedies."

"In another case, many years afterwards, in which the patient, Mrs. V., was cured by another process, and in which I gave the infusion of bark with sulphuric acid, merely to remove the subsequent debility, its exhibition for a single day produced a renewal of the local inflammation. And when scarcely crediting the connexion of circumstances, I repeated the same remedy after the cessation of the symptoms, a precisely similar effect in a few hours followed. After this second trial, I did not consider myself as justified in making a farther attempt, and the patient continued free from relapse." 232.

In a third instance, the patient had begun to take cinchona at the commencement of a very inflammatory attack of this malady, and at the end of ten weeks was precisely in the same state as that in which, under other treatment, he would have been in the first week. "A different plan was adopted, and the patient soon happily recovered."

Dr. Parry thinks that the published cases do not plead very strongly in behalf of this remedy in acute rheumatism. The doses were so small as not to have had much power over the disease, while, on the other hand, fatal symptoms appear to have supervened in some instances under its administration. In his own practice, of 35 years' duration, Dr. Parry never met with an instance of death from acute rheumatism. Dr. Parry does not seem to have been aware of the tendency of acute rheumatism to affect the heart. This translation we consider as one of the main sources of cardiac disease.

"In three cases which I have within these two years attended, and in which the disease had previously run out to a great length, and had either become chronic, or had extremely reduced the constitution of the patient, I have found cinchona produce good effects on the general health; and I greatly doubt whether, under any other circumstances of this malady than those which I have stated, it will, in future experience, be found equally beneficial with other means." 233.

Here our author asks the question, "*What is the difference between rheumatism and gout?*" and from the tenor of his questions and answers one would suppose he did not acknowledge any real difference. In this, we think Dr. Parry is decidedly wrong; for, although it be somewhat difficult to give a specific distinction between the two diseases in a short definition, yet we think that the history, causes, and phenomena are so different as rarely to occasion any difficulty in distinguishing them, even among the least experienced part of the profession. We shall quote this curious passage, however, from our talented author.

"*What is the difference between Rheumatism and Gout?*—1st. Does it consist in the symptoms of the affected parts? Certainly not. In the cases supposed to be distinguishable, the part or parts in both are sometimes swelled, sometimes not swelled, sometimes hot, sometimes cold, sometimes red, sometimes pale.

"2dly. Does it depend on the nature of the pain? No. In both, the pain sometimes exists while the parts are at perfect rest, sometimes occurs almost only when the parts are moved or touched. In both, also, the pain is sometimes alleviated, sometimes increased, by the abstraction of heat; and the converse.

"3dly. Does it depend on the nature of the parts affected?

"Here too there is no just ground of discrimination. Both maladies tend to fix on the neighbourhood of the capsular ligaments of joints.

"If the abstraction of stimulus produces direct debility, and this is the *occasion* of the gout, according to Brown, the actual paroxysm of gout certainly consists in the increased excitement which follows; and, in this respect, what difference is there in the effect between this disease and acute rheumatism, consisting in the undue excitement of parts which have previously undergone direct debility from the application of cold, or, in more philosophical language, from the abstraction of the stimulus of heat? On this theory, what difference ought there to be in the practice in the two cases, the causes and actual state being the same?" 240.

We consider that the causes and actual state of the two diseases are *not the same,* and we are quite confident the treatment must be different to be successful.

XIII. Under the head—"*Gout of the stomach a true inflammation,*" Dr. Parry relates an interesting case of a medical gentleman, some particulars of which we shall here record.

Mr. C. (an eminent surgeon) was 47 years of age. From his youth he had been subject to violent inflammation of the joints "usually called gout," which, for the last twelve years, had attacked him biennially, confining him several months annually to his room. He was a free liver, and late sitter up at night—but took strong exercise on horseback, in the way of his profession through the day. He was liable to indigestion, and, for the last three years, suffered sickness, vomiting, debility, with weak, irregular pulse, and tendency to syncope, whenever the gout left his hands and elbows, however slowly. This did not take place when the gout receded from the lower extremities.

During and after his gouty attacks, he continued his wine, from a pint to a bottle, together with a pretty good allowance of ale.

On the 27th January, 1808, after an attack of gout in the extremities, he began to be sick, and so continued—being unable to take solids, and always uneasy after fluids. He had heartburn and gastrodynia, with tenderness on pressure, and frequent eructations of wind. He quickly ejected the greater part of what he swallowed. At first acid, and ultimately tinged, as if from an admixture of blood. He continued to take wine, usquebaugh, brandy, gout cordials, and other stimulants, till Dr. Parry saw him on the evening of the 3rd of February. He was then very weak, and complained only of sickness, with very little tenderness at the pit of the stomach. The pulse was natural in frequency, though fuller and stronger than usual. Ingesta produced sickness, but not pain of stomach—tongue red at the point, and brown posteriorly—thirsty. —Blisters to the stomach and other parts—purging pills. These measures were wholly unavailing. The symptoms continued without alleviation, and every thing swallowed was brought up. He died on the 8th February.

*Dissection.* There was little or no disease of structure in any part of the body, except in the stomach. This viscus was slightly distended with air—its outside natural—the villous coat was diseased, the whole cardiac portion being more or less inflamed, and presenting dark red patches, of various sizes, having gradually less of interval, till along the great curvature the villous coat was entirely of this colour. The diseased surface, when cut, poured out blood. In the thorax, the lungs were found healthy, the heart loaded with fat.

XIV. The farther we proceed in the perusal of this posthumous volume, the more cause have we for deploring the fatal stroke that arrested the career of its illustrious author. The abruptness of the notes and memoranda—their general want of connexion—their perpetual allusions to cases and names which are no where to be found in the work, render the isolated observations extremely confused and unsatisfactory. Their value is, of course, greatly lessened—and, as we said before, they form merely a granary of rough, unarranged materials, through which we may search a long time for any article we want—and, after all, be disappointed. At other times, the pathologist and practitioner will stumble, as it were by accident, on valuable fragments, which he may turn to use in his daily pursuits or avocations. We shall here introduce an extract more connected in its parts and more legitimate in its conclusions, than is generally the case in this volume.

" *Necessity of certain Determination to the healthy Function of the Brain.*—I. When a certain degree of impulse or momentum of blood takes place in the vessels of the brain, a due excitement, excitability, and balance occur in the external and internal senses, and in the conscious (voluntary) and automatic (tone) motions of voluntary muscles.

" II. If the impulse is somewhat greater, general sensibility is increased, and voluntary power of muscles diminished.

" III. Go on still farther, and one of two things happens.

" 1st. Either partial sensibility increases, and general diminishes, and then voluntary power of muscles increases in a certain proportion to the latter; or,

" 2dly. General sensibility diminishes, and general voluntary power of muscles increases.

" These states II. and III. (1.) (2.), constitute the usual, but not all the states of insanity; because, perhaps, false perceptions, not conclusions of reasoning, or errors of judgment, but actual impressions on the brain, may occur from increased impulse from determination of blood.

" And, perhaps, all these states may arise from other stimuli, as exostoses, ossification, tumors, &c., acting on the brain; but, quære, do they not themselves give occasion to temporary increased determination of blood to the part, or at least concur with it? because in such cases, and in epilepsy from similar causes, the paroxysm is not always permanent. See the remarkable case of Mr. W., and Miss F. May not these exostoses, &c., be effects of long continued determination, and in no degree the causes of epilepsy, &c.?

" It seems as if in the examples which I have pointed out, muscular strength is proportioned, in a certain degree, to diminution of general or nearly general sensibility.

" It is the same locally. What is called weakness, is in fact usually pain or uneasiness in exertion. Thus in chronic rheumatism, &c., a man says his limb is weak, when, on analysing his feelings, and inquiring more minutely, you will find that it is nothing more than that exertion produces such a degree of uneasiness as indisposes him, rather than incapacitates him, for exertion. This, indeed, is often in such cases obvious; for the disease shall be in the joint or capsular ligament, or cellular membrane, which is merely passive; and the muscles, which are the agents, shall be free from disease, and retain their full powers, and yet the man shall assert that he is weak. Give, however, a motive which shall overcome pain, as setting his house on fire, and he shall no longer feel weakness.

"Even in muscles themselves, strength or the capacity of exertion seems usually limited by the sensation of the part. The first symptom of lassitude or weakness is an uneasy feeling in exertion, which indisposes the person to continue it. Rest takes away that uneasiness, and exactly in proportion as it does so, adapts or capacitates the part for renewed exertion. Whatever diminishes sensibility, whether generally or locally, does the same. Thus wine, opium, and tobacco, are well known to produce the first effect, and the abstraction of heat, by the application to the part of cold air or cold water, to produce the latter.

"Even in the case of faintness caused by bleeding, the man who drops his arm from what is called weakness, is, as I have often myself felt, impelled to do so by an undescribable and irresistible sense of uneasiness in the arm itself, which ceases to be felt when the arm is suffered to repose itself." 270.

XV. Immediately succeeding the above extract there are several interesting cases related by Dr. Parry, of determinations of blood to the brain, detailed with more than usual circumstantiality. One or two of these we shall notice.

*Case.* Mrs. C. aged 45 years, became affected in the spring, in the midst of good health, with an herpetic eruption on the left arm, for which mercurials were prescribed. These being continued for a fortnight, a violent salivation ensued and lasted a month, during which the eruption increased. In this period a pain came on in the head, especially in the left side, where it continued, sometimes accompanied by soreness and swelling about the head and neck, but without external inflammation, and constantly attended with fever and loss of appetite. She gradually emaciated. After some time the head got better, and the complaint appeared to be transferred to the left shoulder, which became affected with violent pain and convulsive twitchings. The least motion or touch, from the elbow to the shoulder puts her into agonizing pain. All noise agitates her. She rarely has any sleep.

This patient was directed to be bled, purged, and to take saline medicines with squills. But, a few days after Dr. Parry saw her, she was suddenly seized, one morning, with a violent convulsion, and expired. No examination was made after death.

XVI. *Hydrocephalus.* It does not follow, Dr. Parry observes, that, in disorders of the brain, as, for instance, in insanity, a fulness of the vessels should accompany a preternatural quantity of water in the ventricles. "In fact, the contrary is likely to happen, from the evacuation of the vessels; and we see it actually happen in hydrothorax and other dropsies beginning with inflammation, but of which no signs remain after the fluid has been copiously effused." Here our author introduces a very long case, with the *post mortem* appearances, which we shall greatly abridge.

Mary M. aged seven years, had been accustomed, from the age of three years, to cry out, on first waking, with pain on the left side of the occiput. This symptom went on gradually increasing. One day she had an aggravated attack, and fell into a state of insensibility which lasted some hours. These fits now returned frequently, and she often appeared dull and stupid between them. She went on in this way nearly two years, when, being in better health than usual, she fell down suddenly in a convulsion, and soon expired.

*Dissection.* The vessels of the head and neck were unusually turgid. Dura mater natural. Between it and the pia mater there were about three ounces of serous fluid. The blood was every where fluid, and the vessels of the pia mater were prodigiously distended.

"In this membrane, (pia mater) on the lower side of the left hemisphere of the brain, near the sella turcica, there were several bladders filled with fluid, the largest of which were about the size of a small pea; and others still larger in that part of the pia mater which invested the cerebellum on the right side, immediately under the tentorium. The substance of the brain, and of the right lobe of the cerebellum, was in the natural state; the left lobe of the latter entirely changed, being nearly throughout of a yellow colour, hard, elastic, brittle, in some parts of its substance completely ossified, and, by its surface, firmly adhering to the tentorium. The medulla oblongata had also entirely lost its natural appearance, being precisely similar in structure to the left portion of the cerebellum, except that it was free from ossification. When strongly pressed in any part by the nail, it broke exactly like a piece of boiled gristle. In the ventricles of the brain there were at least four ounces of a clear fluid, which did not coagulate on the application of heat." 301.

The only thing in the symptoms of the above case that indicated disease of an organic nature in the brain, was a tottering in her walk, which continued more or less during the whole of the two years that she was attended by Dr. Parry. It is most strange, however, that, on the very day of her death, she was able "to walk about, eat bread, and drink tea."

We shall conclude the present article with some particulars of an extremely interesting case, detailed with great minuteness by our lamented author. It was a case of chronic inflammation of the membranes of the brain—particularly of the pia mater and tunica arachnoidea.

Lord E———, aged 40 years, a general in the army, accustomed to violent exercise, and who had served in Egypt, became affected with a faltering in his speech, in the middle of the year 1810, without any premonitory symptom. About two months afterwards he became affected with incontinence of urine, especially while walking, accompanied by a heaviness in his lower extremities. These complaints gradually increased, until he was hardly able to totter from one room to another without assistance. He now passed his urine and fæces with little consciousness—became speechless, apparently from mental imbecility, muttering a few words in an unintelligible jargon. Yet he was dressed every day, and took nourishment with appetite, though with little distinction of taste. At times he was affected with sudden fits of shivering and grinding of his teeth.* In June, 1812, he came to Bath. His countenance was pale—he sat almost constantly in a stupid state—his bowels were constipated—his head drooped to one side—he was irritable when spoken to. On the 11th of August he was seized with a strange fit of torpor, in which the food fell out of his mouth. Bleeding, purging, and other remedies, seemed to produce little effect. He continued in a stupid and insensible state till the 15th, when he answered some questions put to him, though imperfectly. From this time, Lord E. began to recover beyond expectation, and, in a few days, he was able to go into an adjoining room, and shortly afterwards to go out in a carriage. His powers of attention and of speech were greatly improved, so that, by the middle of September, he understood perfectly every thing that was said to him, and would often make spontaneous and apposite remarks. He ate and drank well, helping himself to food. Still he complained of his back, and passed his urine and stools unconsciously.

The favourable appearances above described gradually vanished: and, without any apparent cause, Lord E——— relapsed, in the course of two months, into the same stupid state as he

* It ought to have been stated that, in an early period of life, he had experienced a *coup de soleil* in America, and that, three years before the present attack, he was suddenly seized with double vision, which continued three months, and then as suddenly ceased.

had been in when he first came to Bath. In this wretched condition he remained till the month of January, 1813, when he entirely lost the power of speech, though he seemed still to understand the import of questions put to him. He lingered out till the 19th of May, when death freed him from a tiresome and useless existence. The body was opened on the day of his death.

The thoracic and abdominal viscera were perfectly healthy. The cranium was very hard and thick. The veins of the pia mater were gorged with black blood. The membrane itself was considerably thickened, and had a whitish appearance, with some fluid effused between it and the tunica arachnoidea. Numerous red points were seen when the cerebrum was sliced. The whole substance of the brain was exceedingly soft and watery, and this was the condition of the medulla oblongata and all the cerebral nerves. Twelve ounces of fluid were found in the ventricles—eight in the left, and four in the right side. It does not appear that the spinal canal was examined.

Here we must close our present paper, reserving, for another number, our account of the remaining subjects, many of which are of great importance, as, for instance, nervous complaints—hysteria—insanity—epilepsy—apoplexy—paralysis, &c. &c.

We have been greatly surprised, as well as disappointed, at the total want of forwardness for publication in the present memoranda—a want which could not have been anticipated from the expressions made use of in Dr. Parry's first volume. Nor do we see how the younger Parry or any other person, except the original author, could have made an attempt at arrangement of the loose hints, imperfect cases, and insulated fragments, of which these posthumous writings are composed. Although many useful materials will be selected from these volumes by future authors, yet we are not quite sure that a certain degree of unintentional injustice has not been done to the reputation of the illustrious dead, by giving publicity to these posthumous papers in their present condition. At least, such is our own feeling upon the occasion, but we may be wrong. If we are right, the person aggrieved can know nothing of the injury, and the public at large are the gainers.

